# Effect of pig breeding scale on manure resource utilization-The moderating effect based on technology cognition

**DOI:** 10.1371/journal.pone.0314410

**Published:** 2025-01-10

**Authors:** Jiangqi Sun, Zongzheng Liu, Juan Ai, Zhaojiu Chen

**Affiliations:** 1 School of Economics and Management, Jiangxi Agricultural University, Nanchang, Jiangxi, China; 2 Qingdao Institute of Animal Husbandry and Veterinary Science, Qingdao, Shandong, China; Center for Research and Technology Transfer, VIET NAM

## Abstract

The utilization of manure resources is an important measure to promote the development of agricultural green low-carbon cycle and solve the challenges associated with the current large-scale development of the livestock and poultry breeding industry. Based on the survey data of pig farmers in Qingdao, Shandong Province, China, this paper constructs a theoretical analysis framework of pig breeding scale and technical cognition on the utilization behavior of livestock and poultry manure resources of pig farmers. The binary Logit model and the moderating effect model are used to deeply explore the scale effect of breeding scale on the utilization behavior of pig farmers’ manure resources, and the moderating effect of technical cognition on the influence of breeding scale on the utilization behavior of manure resources. First, at the present stage, pig farmers show certain differences in the resource utilization of manure. Due to the differences in the personal characteristics, family characteristics, and breeding characteristics of pig farmers, the influencing factors of resource utilization of pig farmers of different scales are different; Second, the scale of pig breeding has a significant positive promoting effect on the resource utilization of manure, increasing the probability of pig farmers to treat manure, guiding retail and small-scale farmers to moderately expand the scale of breeding, gradually moving to large-scale breeding, realizing centralized management and resource utilization of manure, and reducing the unit cost of manure treatment. Third, technical ease of use has a positive regulatory effect on pig breeding scale and manure resource utilization behavior. When pig farmers perceive that the technology of manure resource utilization is easy to use, they will increase the probability of participating in the resource utilization of manure, reduce the environmental pollution caused by improper disposal of manure, and promote the low-carbon and circular development of livestock and poultry industry. Based on the above findings, this paper aims to provide practical enlightenment for policy makers and researchers to strengthen the environmental governance and sustainable development of livestock industry.

## Introduction

The resource utilization of livestock and poultry manure is the key to ensure the sustainable development of pig breeding industry. China has always been the world’s largest producer and consumer of pork [1]. In 2022, China’s pork production and consumption has accounting for 48.44% and 50.73% of the world’s total pork production and consumption, respectively [2]. It can be seen that the stable development of China’s pig industry is of great significance to food safety, economic development, and social stability in China and the world. As per capita demand for meat continues to increase worldwide, the number of livestock has also increased, increasing livestock manure [3]. Due to the constraints of season, inconvenient application, and other factors, the chain of internal material circulation and energy circulation in agricultural production has been interrupted, resulting in many manure resources becoming major pollution sources [4]. According to statistics, China’s annual output of livestock and poultry manure is as high as 3.8 billion tons, and improper waste disposal has caused nutrient pollution in soil and water [5]. The carbon emissions of the livestock and poultry breeding industry are widely present throughout the prenatal, intrapartum, and postpartum processes. The carbon emissions of end treatment directly affect the rural living environment and agricultural non-point source pollution. In the livestock and poultry breeding industry, carbon emissions from pig breeding are the largest source, especially pig manure, which poses additional risks to the environment and human health [6]. Due to environmental problems, the scale production of livestock and poultry breeding is limited. At the same time, making full use of manure resources is an important basis for sustainable agricultural development, and it is also the focus of the international community, which has aroused extensive discussions in developed and developing countries.

In 2020, the "Opinions on Promoting the High-quality Development of Animal Husbandry" issued by the General Office of the State Council of China stipulates that by 2025, the scale rate of livestock and poultry breeding will reach more than 70%, and the comprehensive utilization rate of livestock and poultry manure will reach more than 80% [7]. In 2010, the standardization demonstration activities of livestock and poultry breeding were carried out to further promote the construction of standardized large-scale livestock and poultry farms, and the standardization and industrialization of large-scale livestock and poultry breeding were realized [8]. Therefore, large-scale breeding is the future development trend and inevitable requirement of the pig industry. The selection of reasonable manure treatment and utilization methods in large-scale farms can not only replace fertilizer with manure to improve the soil and reduce environmental pollution to the greatest extent but also effectively reduce greenhouse gas emissions and take the road of sustainable development [9].

The resource utilization of livestock and poultry manure can be defined as: ’the process of converting livestock and poultry manure into fertilizer, energy or feed through reduction, render harmless and resource treatment [10]. Manure resource utilization construction centered on the return of livestock and poultry manure to the field. The recycling of this product can also reduce the use of high-carbon products by replacing chemical fertilizers with organic fertilizers, which is a beneficial substitute to agricultural resources and can reduce the resource input of the natural environment to the agricultural economic system. It is conducive to comprehensively strengthening the control of agricultural non-point source pollution. It is of great significance to construct a new type of planting-breeding relationship and develop green low-carbon ecological circular agriculture [11]. At the same time, strengthening the end treatment of livestock and poultry manure has also alleviated the pressure on greenhouse gas emissions from animal husbandry to a certain extent [12].

As the most basic micro-subject of pig breeding industry, farm owners are also the most direct executors and beneficiaries of pig manure resource utilization. It is of great significance to explore their resource utilization behavior. At present, the utilization of manure resources in China has entered a stage of in-depth research. The existing literature on the utilization of livestock and poultry manure resources is relatively rich, but most of them carry out research on a certain aspect, but few literatures conduct in-depth systematic analysis of the selection of livestock and poultry manure resource utilization methods based on technical cognition. In order to better promote the process of resource utilization of livestock and poultry manure and ensure the sustainable development of animal husbandry.

In this study, pig breeding scale, technical cognition, and fecal resource utilization behavior were integrated into the same theoretical framework for analysis, and relevant studies using this integrated framework for empirical analysis are relatively rare. At the same time, it is not accurate to study the waste resource utilization of pig farmers by ignoring the scale factor. Within the context of sustainable development goals, it is essential to systematically investigate the influence mechanisms of pig scale breeding and technical cognition on the utilization behavior of main manure in pig farms.

## Theoretical analysis and research hypothesis

### Analysis of the influence of pig breeding scale on the utilization of manure resources

With the increasing demand for pig breeding, the scale of breeding is also expanding. As a rational economic man, pig farmers will use less cost expenditure to obtain high-yield manure treatment methods when carrying out manure resource utilization. As the main executive body and the most basic micro decision-making unit of livestock and poultry manure treatment, pig farm owners play a key role in promoting the pollution control of livestock and poultry breeding. Chen Feifei and other studies have shown that the cost of pig manure treatment in small-scale pig farms is the highest, and it is highly dependent on government subsidies [13]. Because small-scale breeding generally does not reach the optimal breeding scale, and the level of mechanization is low, the labor cost is high, resulting in high cost of pig manure treatment. Due to the small scale and small capital of scattered farmers, it is easy for them to withdraw from resource utilization because of the shortage of funds when dealing with the continuous increase in the cost of pig manure treatment. Due to the existence of scale effect, the production cost of large-scale farms is lower, and the breeding efficiency is higher than that of free-range households [14]. Through the expansion of pig breeding scale, the cost of manure treatment can be directly reduced by optimizing the allocation of production factors, thus promoting the utilization of manure resources. Based on the above analysis, this paper puts forward the research hypothesis 1.

**H1**: The scale of pig breeding has a positive effect on the utilization of manure resources, and pig farmers who carry out large-scale breeding are more inclined to the utilization of manure resources.

### Analysis of the moderating effect of technical cognition on the influence of pig breeding scale on the utilization behavior of manure resources

Technical ease of use is an important factor affecting the utilization of manure resources by farm owners. The manure treatment of large-scale livestock and poultry breeding in China is still dominated by fertilizer utilization mode [15]. However, the education level of pig farmers is generally low, and their production and operation decisions are often affected by production skills and knowledge. The cognition of the difficulty of operation of manure treatment technology is the key factor affecting their technology adoption behavior. Large-scale farming is usually closely related to modern equipment and production technology [16]. On the one hand, pig farms with large enough scale have strong technological innovation ability; even if there is no innovation ability in general-scale pig breeding, it is easier to accept and adopt new technologies than free-range households [17]. The choice of waste resource utilization methods in pig farms is affected by technical cognition. Perceived ease of use refers to the perception of farmers on the convenience of using livestock and poultry manure resource utilization, which reflects the feasibility of farmers to implement livestock and poultry manure resource utilization. For farmers, the resource utilization behavior of livestock and poultry farming may take time and effort and may be somewhat difficult compared to direct emissions. However, if farmers feel that the technology is simple and easy to master, the probability of participating in resource utilization behavior may be greatly increased [18]. The more pig farmers feel that this waste treatment technology is more convenient and easy to use, the higher the possibility of waste resource utilization, the more conducive to improving the degree of waste resource utilization, and thus promoting the green and low-carbon development of pig farming. Zhu Run et al. found that the lack of technical cognition will enhance the uncertainty of large-scale farmers’ utilization of pig manure resources, resulting in fear of difficulties [19]. For pig farmers, the resource utilization of livestock and poultry manure may take time, energy and money, which may be slightly more difficult than random treatment. If the pig farm owners feel that the operation is simple and easy to master in technology, the probability of their participation in resource utilization will be greatly increased. Zhang Jiaqi and other researchers have also shown that the perceived ease of use of farmers will increase the likelihood of their behavior [20]. Based on the above analysis, this paper puts forward the research hypothesis 2.

**H2**: The technical ease of use has a positive regulatory role in the scale of pig breeding affecting the behavior of manure resource utilization, that is, the higher the degree of technical ease of use, the more conducive to the utilization of manure resources by pig farmers.

## Methodology

### Data source

Shandong Province is a major pig-raising province in China, and the pig industry occupies a pivotal position in the animal husbandry of the whole province. With the continuous improvement of the scale, intensification and modernization of animal husbandry in Qingdao, the development system of modern animal husbandry with urban high-end characteristics has been preliminarily constructed.

Before data collection, the questionnaire was approved by the University’s Ethics Committee (Jxaull-20230606) and does not contain any sensitive information. Besides, the participants were informed that involvement was anonymous and completely voluntary because they could opt-out at any time without consequences. The data employed in this paper stem from the questionnaire survey data collected in Qingdao pig farms in China during the period from July 2023 to September 2023. To ensure the representativeness of the research objects and the reliability of the research results, four districts and three cities in Qingdao with significant pig waste disposal tasks were selected, namely Laoshan District, Chengyang District, Huangdao District, Jimo District, Jiaozhou City, Pingdu City and Laixi City. Among them, Laoshan District pertains to the main urban area, where the urban type modern animal husbandry system has been established; Chengyang District, Huangdao District, Jimo District and Jiaozhou City belong to the suburbs and outer suburbs of Qingdao city. They constitute the main distribution areas of the secondary and tertiary industries in Qingdao City. Pingdu City and Laixi City are the main agricultural and animal husbandry distribution areas of Qingdao. In recent years, along with the rapid advancement of the livestock and poultry industry, a considerable amount of livestock and poultry feces are discharged untreated, which renders the issue of livestock and poultry feces pollution highly sensitive within the livestock sector, and the research efforts regarding this problem are relatively challenging. In this study, stratified and random sampling questionnaires were used to obtain survey data. 2 townships were randomly selected in Jiaozhou City, Pingdu City and Laixi City, 3 villages were identified in each township, and 10 pig farmers were investigated in each village. 2 townships were randomly selected in Laoshan District, Chengyang District, Huangdao District and Jimo District, and 3 villages were identified in each township. 5 pig farmers were surveyed in each village, and a total of 300 pig farmers were sampled. The primary participants targeted by the survey were pig farmers, and interviews were conducted on a one-to-one basis. Following the screening and processing of missing values, the number of valid questionnaires amounted to 284. The response rate achieved was 97.93%.

### Variable selection and descriptive statistics

#### 1. Variable being explained

The explained variable of this objective is whether the pig farm has carried out environmentally friendly manure treatment, that is, the resource utilization of manure. What is the way you deal with pig manure in the questionnaire? Ask questions, the answer option is: ’A discarded at will; B returning to the field for utilization; C composting fermentation; D make biogas; E to make organic fertilizer. For the answer to this question, random discarding is defined as non-resource utilization, that is, ’no’ is assigned to ’0’, and other options are defined as resource utilization, that is, ’yes’ is assigned to ’1’. Through the analysis of the sample distribution of pig farm owners’ utilization of manure resources, as shown in Table 1, at this stage, pig farm owners show different characteristics in the utilization of manure resources, and large-scale farms have the highest degree of utilization of manure resources, accounting for 19.72%. Followed by medium-sized farms accounted for 14.79%; the lowest proportion of manure resource utilization in small-scale farms was 6.69%.

**Table 1:** Resource utilization of piggery manure.

Breeding scale	Retail	Proportion	Small scale	Proportion	Medium scale	Proportion	Large scale	Proportion
Non resource utilization	40	14.08	28	9.86	45	15.85	34	11.97
Resource utilization	20	7.04	19	6.69	42	14.79	56	19.72

#### 2. Core explanatory variables

**Fig 1:**
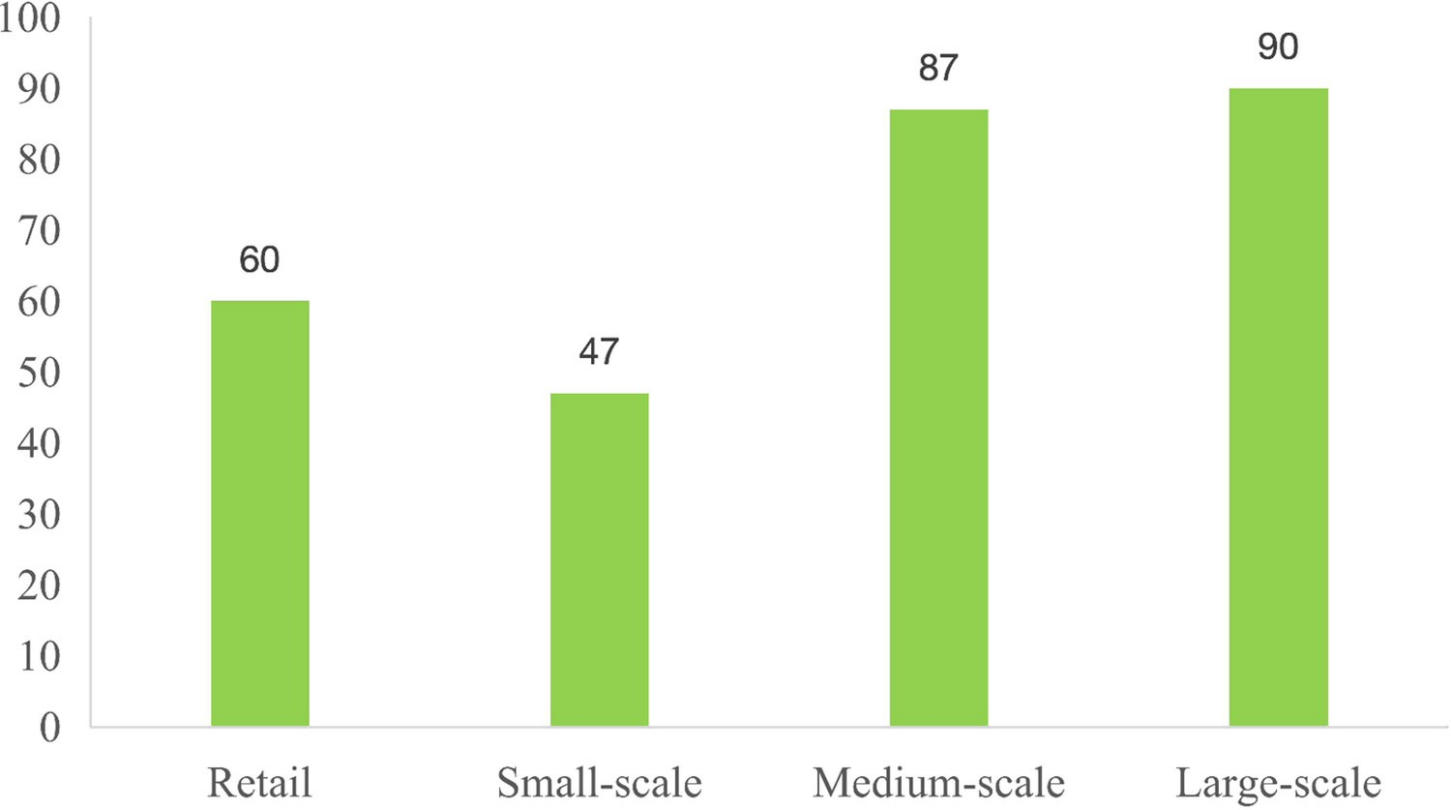
Scale of livestock and poultry farms. Data source: Collated according to survey data.

The purpose of this objective is to explore the impact of pig breeding scale on the utilization of manure resources, and there is no uniform standard for the division of breeding scale in the existing literature. In this paper, according to the ’national agricultural product cost-benefit data compilation’ regulations, the scale of pig breeding is divided into: retail (annual sales volume≤30), small-scale (30<annual sales volume≤100), medium-scale (100<annual sales volume≤1000), large-scale (annual sales volume>1000). According to the data collected from the survey presented in Fig 1, the pig breeding in Qingdao is mainly based on medium-scale breeding and large-scale breeding.

#### 3. Regulated variable

Based on Li Wenhuan’s research, this objective selects the technical ease of use in technical cognition as a regulatory variable, and uses the difficulty of pig farmers in using livestock and poultry manure resource utilization treatment technology as a characterization [21]. From the sample distribution of the ease of use of pig manure treatment technology by pig farm owners in Table 2, it can be seen that the pig farm owners believe that the pig manure treatment technology is very difficult and difficult to account for 43.31% of the total number of pig farms, indicating that most pig farm owners believe that the current manure treatment technology is not easy to use, so that the pig farm owners with low education level are hindered in the process of manure resource utilization.

**Table 2:** Sample distribution of technology ease of use.

Index	Very difficult	More difficult	General	Relatively simple	Very simple
Sample size	59	64	60	51	50
Proportion	20.77	22.54	21.13	17.96	17.61

Data source: According to the survey data.

#### 4. Control variable

Based on the existing research, this objective takes the individual characteristics, family characteristics, breeding characteristics, cognitive characteristics and government policy characteristics of farm owners as control variables. Among them, the individual characteristics of farm owners select variables such as age, education level, and whether to serve as village cadres; family characteristics select the number of non-agricultural labor force; breeding characteristics select variables such as breeding years and breeding income; cognitive characteristics Select the cognition of the impact of livestock and poultry manure on human health and the cognition of livestock and poultry breeding environmental protection; the government policy characteristics select variables such as government propaganda and government subsidies. The specific variable description is shown in Table 3.

**Table 3:** Variable description and descriptive statistics (N = 284).

Type	Variable	Variable measurement	Mean value	Standard deviation
Variable being explained	Waste recycling	1 = Yes, 0 = No	0.482	0.501
Core explanatory variables	Breeding scale	1 = Annual sales volume≤30head, 2 = 30<Annual sales volume≤100head, 3 = 100<Annual sales volume≤1000head, 4 = Annual sales volume>1000head	2.729	1.122
Regulated variable	Usability of technology	1 = Very difficult, 2 = More difficult, 3 = general, 4 = relatively simple, 5 = Very simple	2.891	1.391
Control variable	Age	The age of the head of the farmer (years)	48.282	8.145
	Education degree	1 = Primary school and below, 2 = junior high, 3 = senior high, 4 = college or higher	2.475	1.091
	Is he a village cadre	1 = Yes, 0 = No	0.472	0.500
	Number of non-agricultural labour force	1 = 1~2, 2 = 3~4, 3 = 5~6, 4 = 7~8, 5 = 8higher than	3.187	1.423
	Breeding years	1 = Within 1 year, 2 = 1~3year, 3 = 3~5year, 4 = 6~8year, 5 = 8higher than	3.134	1.398
	Annual income of breeding	1 = 1 million and below, 2 = 100~300million yuan, 3 = 300~500million yuan, 4 = 500More than ten thousand yuan	2.563	1.121
	Cognition of the impact on human health	1 = There is no impact at all, 2 = There is a bit of influence, 3 = general, 4 = has great influence, 5 = has great impact	3.423	1.274
	Cognition of environmental protection of livestock and poultry breeding	1 = Very not understand, 2 = Not very understanding, 3 = general, 4 = Comparative understanding, 5 = Very understanding	2.430	1.194
	Propaganda of government	1 = Yes, 0 = No	0.391	0.489
	Government subsidies	1 = Yes, 0 = No	0.489	0.501

### Model setup

Since the utilization of manure resources is a binary variable, the binary Logit model is selected to estimate it with reference to Meraner M’s research [22, 23]. In order to test the impact of large-scale pig breeding on the utilization of manure resources, the model is established as follows:

$$Y=\alpha_{0}+\alpha_{1}Scale+\alpha_{2}Technique+\sum \beta_{i}X_{i}+\varepsilon_{1}$$ (1)

In order to estimate the moderating effect of technical ease of use in the influence of pig breeding scale on the behavior of pig farm owners’ manure resource utilization, referring to Wen Zhonglin’s research [24], a regression model including the interaction term of breeding scale and manure resource utilization was established on the basis of formula (1). The model is as follows:

$$Y=\alpha_{0}+\alpha_{1}Scale+\alpha_{2}Technique+\alpha_{3}Scale\times Technique+\sum \beta_{i}X_{i}+\varepsilon_{2}$$ (2)

In Eqs (1) and (2), Y refers to the manure resource utilization behavior of farm owners, Scale represents the scale of pig breeding, Technique represents the ease of use of technology, Scale Technique is the interaction term between the scale of breeding and the ease of use of technology, Xi is a series of control variables, a_1_, a_2_, a_3_ and *β*_*i*_ are estimated coefficients, a_0_ is a constant term, *ε*_1_ and *ε*_2_ is a random error term.

## Discussion

### The influence of breeding scale and technical cognition on the utilization of manure resources

In this paper, stata16.0 software was used to analyze the binary Logit model of pig farm data. Model 1 verified the effect of the breeding scale on the resource utilization behavior of pig farmers’ manure. Model 2 verifies the impact of technical ease of use on pig farmers’ waste resource utilization behavior. Model 3 verified the combined effect of farming scale and technical ease on the resource utilization behavior of pig farmers’ manure. Based on Model 3 data. Model 4 calculates the marginal effects of each variable. The empirical results are shown in Table 4.

**Table 4:** The main effects and regulatory effects of breeding scale and technical ease of use on the utilization of manure resources.

Variable	Model group 1	Model group 2	Model group 3	Model group 4	Model group 5
Breeding scale	0.452***	-	0.433***	0.083***	0.456***
	(0.128)	-	(0.130)	(0.024)	(0.133)
Usability of technology	-	0.264***	0.243**	0.049**	0.245**
	-	(0.099)	(0.101)	(0.020)	(0.104)
Breeding scale×Usability of technology	-	-	-	-	0.261***
	-	-	-	-	(0.093)
Age	-0.006	0.007	-0.003	0.000	-0.001
	(0.017)	(0.017)	(0.017)	(0.003)	(0.018)
Education degree	0.236**	0.263**	0.281**	0.054**	0.296**
	(0.126)	(0.127)	(0.131)	(0.025)	(0.133)
Is he a village cadre	0.811***	0.832***	0.833***	0.166***	0.805***
	(0.276)	(0.273)	(0.279)	(0.054)	(0.283)
Number of non-agricultural labour force	-0.187*	-0.199**	-0.182*	-0.036*	-0.187*
	(0.097)	(0.095)	(0.098)	(0.019)	(0.100)
Breeding years	-0.221**	-0.228**	-0.231**	-0.047**	-0.246**
	(0.100)	(0.098)	(0.100)	(0.019)	(0.103)
Annual income of breeding	0.341***	0.342***	0.334***	0.064***	0.346***
	(0.125)	(0.124)	(0.126)	(0.024)	(0.129)
Awareness of environmental policy	0.308***	0.275**	0.276**	0.056**	0.305**
	(0.116)	(0.116)	(0.119)	(0.023)	(0.122)
Cognition of human health	0.242**	0.185*	0.225**	0.041*	0.239**
	(0.111)	(0.108)	(0.113)	(0.021)	(0.115)
Propaganda of government	0.137	0.238	0.125	0.024	0.109
	(0.282)	(0.277)	(0.287)	(0.055)	(0.291)
Government subsidies	0.668**	0.607**	0.682**	0.132**	0.707**
	(0.276)	(0.271)	(0.279)	(0.054)	(0.284)
Constant term	-3.536***	-3.471***	-4.315***	-	-4.588***
	(1.141)	(1.144)	(1.209)	-	(1.244)
Fake R2	0.177	0.162	0.192	-	0.214
LR chi-squared	69.78***	63.96***	75.68***	-	84.05***
Sample size	284	284	284	284	284

Note:

*, **, *** are significant at the statistical level of 10%, 5% and 1%respectively, and the standard error is in the brackets. The same below.

The chi-square test statistics of comprehensive analysis model 1, model 2 and model 3 are all significant at the statistical level of 1%, indicating that the fitting effect of the regression model is good, so it has the significance of further analysis. At the same time, the empirical results show that the significance of each variable in the model is basically consistent, which indicates that the estimation results of the model are robust.

Through the analysis of the above empirical results, the scale of pig breeding has a significant positive impact on the utilization behavior of manure resources, which promotes the probability of pig farmers’ utilization behavior of manure resources to increase by 8.3%, and is significant at the level of 1%, as shown in Table 4 Model 4. That is to say, large-scale pig breeding has a promoting effect on the utilization of manure resources by reducing the unit cost of manure treatment, which is verified by hypothesis 1 [25]. In addition, at the statistical level of 5%, the ease of usability of technology has a positive impact on the waste resource utilization behavior of pig farmers, that is, the higher the usability of technology, the more inclined pig farmers are to waste resource utilization behavior. As shown in Model 4 of Table 4, the probability of pig farmers’ waste resource utilization behavior increased by 4.9% with each increase of one unit of technology ease usability. This means that the higher the ease of perception of technology by pig farmers, the more conducive to improving the level of waste resource utilization.

In terms of control variables, the education level of pig farmers has a significant positive effect on the utilization of manure resources, and it is significant at the level of 5%, that is, the higher the education level of pig farmers, the greater the possibility of manure resource utilization. Considering that it is difficult for pig farmers to match the utilization of manure resources by changing their education level in the short term, it is necessary for manure treatment technology to be easily used to effectively make up for the lack of labor quality. At the statistical level of 1%, whether they are village cadres or not and the annual income of breeding have a positive and significant impact on the resource utilization behavior of manure of pig farmers, that is, pig farmers who are village cadres and have higher annual income of breeding are more inclined to resource utilization of manure. At the statistical level of 10%, the number of non-agricultural labor force negatively affects the manure resource utilization behavior of pig farmers, that is, the higher the number of non-agricultural labor force, the lower the possibility of manure resource utilization of pig farmers. It may be because the resource utilization of livestock and poultry waste is a typical labor-intensive production activity, and the demand for the labor force is high. Therefore, pig farmers with a small number of labor forces will not carry out waste resource utilization [26]. The years of livestock and poultry breeding have a significant negative impact on the utilization of manure resources. With the increase of the breeding years of pig farmers, most pig farmers will use livestock and poultry manure based on their breeding experience, and the acceptance of new manure treatment technology is not high.

### Analysis of moderating effect based on technical cognition

According to the previous theoretical analysis, technical ease of use has a regulatory effect on the impact of pig farming scale on the utilization of manure resources. The scale of pig farming has different effects on the utilization of manure resources due to different technical ease of use. Based on this, this paper uses the interaction term to verify whether the ease of use of technology has a moderating effect on the impact of pig breeding scale on the utilization behavior of manure resources.

As shown in Table 4, model 5 adds an interaction term between breeding scale and technical ease of use to verify the regulatory role of technical ease of use in the utilization of manure resources by breeding scale on the basis of model 3. Considering that the model may have multicollinearity problems, this paper takes a centralized approach to the two variables of breeding scale and technical ease of use in the study. Subsequently, the cross multiplication of the centralized farming scale variable and the centralized technical ease of use variable is performed to generate an interaction term, which is included in Model 5.

The results show that in Model 5, the technical ease of use and the interaction term of breeding scale and technical ease of use have a positive and significant impact on the utilization of manure resources by pig farmers. This shows that, first, technology ease of use has a significant moderating effect on the impact of breeding scale on the utilization behavior of manure resources; second, the interaction coefficient is positive, which is the same as the main effect that the scale of breeding has a positive impact on the utilization of manure resources, indicating that there is a mutually reinforcing relationship between the two, that is, the simpler the piggery owners perceive the manure treatment technology, the more conducive to the utilization of manure resources, research hypothesis 2 is verified.

### Robustness test

#### 1. Replacement model method

The replacement model method is a common processing method widely used in self-selection problems, especially for causal inference of sampled sample data. In order to test the reliability of the research conclusions, it is necessary to re-validate the impact of breeding scale and technical ease of use on the resource utilization behavior of main manure in pig farms. The 284 data are regressed using the binary Probit model again. The model also passes the significance test. Compared with Table 4, the symbols of the coefficients in Table 5 do not change. Based on the above facts, we draw the following conclusions: The results of this study are not affected by selection bias, and the regression results of the model are stable and reliable.

**Table 5:** Replacement model method.

Variable name	Model group 6	Model group 7
Breeding scale	0.259*** (0.076)	0.269*** (0.078)
Usability of technology	0.153** (0.060)	0.152** (0.062)
Breeding scale×Usability of technology	-	0.153*** (0.054)
Control variable	Controlled	Controlled
Pseudo R-squared	0.194	0.214
Observed value	284	284

In Model 6, the scale of pig farms has a positive impact on the utilization of manure resources by pig farmers, and is significant at the level of 1%. That is to say, the higher the scale of pig farms, the more conducive to promoting the utilization of manure resources by pig farmers. The results are consistent with the results of model 3. In model 7, the coefficient direction and significance degree of variable breeding scale and technical ease of use are consistent with model 5. That is, the interaction between breeding scale and technical ease of use has a positive impact on the resource utilization behavior of pig farms, and it is significant at the level of 1%. This shows that the results of this study are consistent with the previous theoretical viewpoints, which further verifies the accuracy of the previous conclusions.

#### 2. Re-delineating the standard of pig farmers’ breeding scale

According to the classification standard of ’National Agricultural Product Cost-Benefit Data Compilation’, combined with the actual situation of Qingdao City, the pig farmers with more than 30 pigs per year are defined as scale pig farms. However, the basis for the division of breeding scale in different regions is not the same, and there are also differences in academic circles. In view of this, the text selects samples according to different pig breeding scale definition standards, and uses the benchmark model estimation results for robustness testing.

This paper refers to the definition standard of Pan Dan [27], and defines the pig farmers with 50 or more pigs per year as scale farmers, and screens the samples. The regression results are shown in Table 6. Through comparison, it can be seen that the regression results after re-screening the samples according to the two definition criteria are consistent with the regression results of the benchmark model.

**Table 6:** Robustness test: Based on the definition criteria of different scale pig farmers.

Variable name	Model group 8	
	Marginal effect	Standard error
Breeding scale	0.097**	(0.040)
Usability of technology	0.071***	(0.020)
Control variable	Controlled	
Pseudo R-squared	0.206	
Observed value	224	

## Conclusion and policy recommendations

### Conclusions

Based on the micro-survey data of 284 pig farmers in Qingdao, Shandong Province, combined with theoretical analysis and empirical test, this paper discusses the influence of pig breeding scale on the utilization of manure resources and the adjustment effect of technical ease of use in the two. Based on the above empirical analysis, this paper draws the following conclusions: First, at the present stage, pig farmers in Qingdao show a certain trend of differentiation in the utilization of manure resources. Due to differences in personal characteristics, family characteristics and breeding characteristics of pig farmers, there are differences in the influencing factors of resource utilization of pig farmers of different scales. Second, the scale of pig breeding has a significant positive promoting effect on the resource utilization behavior of manure, which increases the probability of the resource utilization of manure by pig farmers, guides retail and small-scale farmers to moderately expand the scale of breeding, and gradually transition to large-scale breeding, so as to achieve centralized management and resource utilization of manure, and reduce the unit cost of manure treatment. Third, technical ease of use has a positive regulatory effect on pig breeding scale and manure resource utilization behavior. When pig farmers perceive that the technology of manure resource utilization is easy to use, they will increase the probability of participating in the resource utilization of manure, reduce the environmental pollution caused by improper disposal of manure, and promote the low-carbon and circular development of livestock and poultry industry.

### Policy proposal

Based on the above research conclusions, in order to reverse the situation from "want me to recycle" to " I want to recycle," this paper puts forward the following suggestions to promote the resource utilization behavior of pig farmers’ manure. First of all, we should accelerate the promotion of pig scale breeding and promote the development of small and medium-sized breeding enterprises. Through policy encouragement and financial support, we will guide retail households and small and medium-sized pig farms to appropriately expand the scale of breeding, gradually transition to large-scale breeding, realize the centralized treatment and resource utilization of livestock and poultry manure, and reduce the unit cost of manure treatment. Secondly, according to the technical cognition degree of pig farmers, according to the household conditions, classified management. Focus on the promotion of applicable technologies coupled with local resource endowment conditions, so as to improve the perceived ease of use of pig breeding waste resource utilization technology by pig farm owners, and then improve the technology adoption behavior of livestock and poultry breeding waste resource utilization. Finally, improve the livestock and poultry manure treatment infrastructure, and cultivate high-quality pig farmers. Strengthen the training of relevant knowledge lectures for pig farmers to improve the knowledge level of pig farmers. Make up for the lack of education level of pig farmers in the early stage, so that they are easier to accept new technologies, and easier to use waste resources.

### Limitations

Although the research results of this study have practical significance, there are still some limitations, which provide a direction for further exploration. First of all, the problem of livestock and poultry manure pollution has certain sensitivity in breeding enterprises, and the research on this problem is relatively difficult. This paper only issued a questionnaire to the areas with heavy pig breeding workload in Qingdao City, Shandong Province. The understanding of the overall livestock and poultry breeding situation in Qingdao City is limited and needs to be further explored. Secondly, the survey uses binary variables, and the discussion on the utilization behavior of manure resources in farms is not detailed enough. However, future research can refine the utilization behavior of manure resources into more types, so as to more comprehensively understand the impact of changes in livestock scale on the utilization behavior of manure resources.

## Supporting information

S1 File Data information. (PDF)
