# Supplementary material for: Effect of pig breeding scale on manure resource utilization-The moderating effect based on technology cognition
Source: PLoS One. 2025 Jan 10;20(1):e0314410. doi: 10.1371/journal.pone.0314410 (PMC11723535; doi:10.1371/journal.pone.0314410)
Supplement: S1 File — (PDF) [file pone.0314410.s001.pdf]

Waste recycling	Age	Education degree	Is he a village cadre	Number of non-agricultural labour force
0	40	3	1	5
0	56	2	1	4
0	56	1	0	3
0	50	1	1	4
0	57	4	0	5
0	40	3	0	4
0	56	3	0	2
0	55	1	1	3
0	49	1	0	3
0	52	2	1	2
0	53	3	1	5
0	38	3	1	5
0	52	4	0	5
0	63	1	0	5
0	55	4	1	2
0	50	1	0	4
0	46	2	1	2
0	36	2	0	3
0	45	1	1	5
0	38	2	1	4
0	36	4	0	5
0	53	1	0	5
0	47	4	0	4
0	50	3	0	2
0	51	3	1	2
0	48	3	0	2
0	51	1	1	5
0	39	1	1	1
0	60	3	0	4
0	42	2	0	4
0	48	2	0	3
0	47	2	0	5
0	54	1	1	5
0	65	2	0	3
0	47	3	1	2
0	53	2	0	3
0	60	1	0	5
0	50	3	0	5
0	52	4	0	1
0	51	3	1	3
0	51	2	0	4
0	41	2	1	5
0	35	1	0	2
0	37	3	0	2
0	60	3	1	5

0	38	2	0	2
0	56	1	1	4
0	44	3	0	4
0	50	1	1	5
0	60	1	0	3
0	58	1	0	4
0	31	2	0	4
0	49	2	0	2
0	59	1	0	3
0	54	3	0	5
0	36	1	0	2
0	45	4	0	3
0	35	4	0	2
0	50	4	0	2
0	42	3	1	3
0	59	3	0	4
0	48	2	0	3
0	49	1	1	3
0	55	2	1	2
0	57	1	1	4
0	44	1	1	4
0	42	3	0	3
0	44	3	1	4
0	40	2	0	2
0	36	4	1	2
0	37	1	0	4
0	52	2	1	3
0	49	3	0	3
0	48	1	0	2
0	26	1	0	2
0	62	3	0	2
0	46	4	1	4
0	60	4	1	2
0	49	3	0	1
0	46	3	0	4
0	53	1	0	5
0	42	2	0	4
0	51	2	0	5
0	46	2	1	4
0	37	2	0	4
0	44	4	0	5
0	50	2	1	1
0	39	1	0	2
0	58	2	1	4
0	47	1	1	5
0	57	1	0	4

0	59	1	0	3
0	38	2	1	4
0	60	4	0	3
0	43	4	0	3
0	38	3	0	5
0	55	2	0	3
0	53	2	1	5
0	59	3	0	4
0	47	1	0	2
0	51	2	0	5
0	57	2	0	5
0	36	4	0	4
0	41	3	1	4
0	59	3	0	5
0	58	3	0	4
0	40	3	0	3
0	47	2	1	4
0	52	3	1	1
0	30	2	1	5
0	38	3	1	1
0	46	4	1	4
0	49	2	0	4
0	49	3	0	5
0	57	1	0	2
0	42	2	1	1
0	63	3	0	2
0	44	3	0	4
0	41	2	0	2
0	36	3	1	4
0	55	3	0	3
0	40	4	1	1
0	53	3	1	1
0	51	2	0	3
0	39	3	0	2
0	64	4	0	5
0	56	1	0	2
0	41	1	1	1
0	46	3	0	4
0	52	4	0	2
0	40	3	0	4
0	50	4	0	5
0	53	1	0	5
0	55	4	1	5
0	59	1	1	4
0	35	2	0	2
0	36	2	1	1

0	58	4	1	4
0	54	1	0	5
0	55	3	0	3
0	59	2	0	5
0	40	4	0	4
0	56	1	1	5
0	27	1	0	2
0	44	2	1	4
0	41	1	1	3
0	36	1	1	1
1	38	4	1	1
1	48	2	1	1
1	41	4	1	1
1	54	2	1	1
1	58	2	0	4
1	57	3	0	1
1	46	4	0	3
1	40	2	0	4
1	49	4	0	1
1	44	3	1	3
1	49	2	1	4
1	49	4	1	1
1	51	2	0	4
1	41	4	1	5
1	61	4	0	4
1	47	3	0	2
1	39	3	0	5
1	54	2	1	2
1	38	1	1	4
1	36	1	0	1
1	57	4	1	1
1	45	4	0	1
1	55	2	0	5
1	40	1	0	4
1	44	4	0	1
1	52	3	0	4
1	44	4	1	4
1	50	4	0	1
1	26	2	0	5
1	46	2	0	3
1	38	1	1	3
1	39	1	0	1
1	55	4	0	2
1	58	2	1	1
1	48	1	0	5
1	38	4	0	2

1	49	3	1	4
1	55	2	0	5
1	36	3	0	5
1	38	1	1	1
1	58	3	1	3
1	46	3	1	3
1	45	2	1	2
1	40	3	1	4
1	51	4	1	1
1	44	1	1	5
1	45	3	1	2
1	40	3	1	4
1	45	4	0	4
1	57	1	1	5
1	42	2	1	5
1	55	4	1	2
1	50	3	1	3
1	54	3	1	3
1	47	1	1	5
1	53	4	1	5
1	54	2	1	5
1	51	2	0	1
1	37	2	0	1
1	55	2	0	1
1	41	4	1	4
1	39	3	1	4
1	46	1	0	2
1	57	2	0	1
1	50	2	0	4
1	45	2	0	1
1	54	4	0	4
1	47	3	1	2
1	58	2	0	4
1	40	1	1	5
1	53	2	1	2
1	47	4	1	5
1	36	1	1	4
1	52	2	1	1
1	42	3	0	3
1	54	1	0	5
1	47	2	0	5
1	56	3	1	4
1	58	3	1	4
1	42	2	1	4
1	58	1	0	3
1	60	2	1	2

1	47	3	0	5
1	47	4	1	3
1	41	4	1	1
1	37	2	1	5
1	53	2	0	4
1	58	2	1	3
1	54	4	1	5
1	47	4	0	2
1	42	2	1	4
1	35	1	1	3
1	51	4	1	5
1	29	4	1	3
1	57	4	1	2
1	59	4	1	2
1	43	2	1	3
1	57	4	0	1
1	45	4	0	2
1	43	2	0	2
1	54	3	0	1
1	52	1	1	5
1	48	2	1	3
1	49	2	0	2
1	50	1	0	5
1	55	4	0	1
1	40	4	1	3
1	48	3	0	1
1	45	1	1	3
1	63	3	1	5
1	57	4	1	3
1	58	4	1	5
1	36	1	0	3
1	49	4	1	5
1	49	3	0	1
1	56	3	0	3
1	60	1	0	1
1	57	2	1	1
1	51	4	0	4
1	67	2	1	1
1	36	3	1	4
1	48	4	1	5
1	49	3	1	3
1	55	1	0	3
1	51	4	0	4
1	50	1	1	1
1	60	4	1	1
1	45	3	1	1

1	42	3	1	5
1	53	2	0	5
1	57	1	1	5
1	59	1	1	1
1	42	4	1	1
1	38	3	0	2
1	65	1	0	1
1	32	3	1	5
1	59	1	1	2

Breeding years	breeding scale	Annual income of breeding	Usability of technology
5	4	4	3
4	4	3	2
5	2	1	3
2	1	1	1
4	2	3	4
2	2	2	2
5	2	4	4
4	2	1	2
5	3	1	1
5	2	1	2
2	3	4	1
3	3	2	4
4	1	3	2
4	3	1	2
3	4	3	1
1	1	4	1
5	3	1	2
4	1	4	4
5	1	1	4
1	3	1	1
1	4	1	2
4	2	1	1
3	2	2	1
4	4	4	1
4	3	2	4
5	1	1	1
5	4	3	2
4	1	1	3
1	4	2	2
3	3	2	2
4	3	2	3
1	1	2	4
1	3	3	5
3	1	1	1
2	4	2	3
1	4	4	1
5	2	1	3
3	3	4	2
5	2	3	2
3	1	3	5
3	3	1	3
3	3	2	3
5	1	4	5
4	4	1	1
2	4	3	1

5	2	2	5
3	4	2	5
5	1	2	2
3	1	2	1
3	2	2	3
3	3	1	5
1	1	1	2
4	2	3	1
5	4	3	4
2	3	1	1
1	2	4	5
3	1	1	1
2	2	4	4
4	1	2	2
4	3	3	1
2	4	1	2
3	4	1	1
3	2	3	4
3	1	1	2
3	4	4	4
3	1	3	5
4	1	1	5
5	3	2	5
5	1	3	4
2	2	1	1
4	4	2	3
2	3	3	2
2	1	4	4
4	3	4	2
5	4	1	4
2	1	4	2
2	3	1	3
3	3	4	1
4	3	1	3
4	3	3	3
2	3	4	3
4	1	3	1
5	3	4	1
2	2	2	3
5	4	1	5
5	4	2	3
4	1	1	3
4	3	4	3
2	3	3	1
1	1	1	3
5	3	4	1

3	4	3	3
3	1	1	2
3	2	3	3
1	3	3	2
2	1	2	3
4	4	1	2
5	4	1	5
1	3	3	5
3	1	1	1
2	1	3	4
4	3	1	2
3	2	1	1
3	3	4	5
4	3	3	2
5	3	3	2
5	1	4	3
3	1	4	1
3	1	1	1
4	3	3	3
3	3	1	4
3	3	3	1
5	4	4	3
3	2	1	1
4	4	4	2
4	3	3	1
1	4	4	1
4	2	3	1
1	4	3	4
5	1	3	4
5	3	1	2
4	2	2	2
2	4	2	2
4	3	2	2
5	4	1	4
5	4	4	4
5	4	2	1
2	2	3	4
5	1	2	4
4	2	1	1
1	1	2	5
4	1	4	5
4	2	1	5
1	3	3	5
3	1	1	4
3	2	3	1
3	4	2	2

5	3	3	5
3	4	2	1
4	4	2	1
4	1	1	3
1	3	2	3
4	1	2	4
1	1	1	4
5	2	3	3
5	3	2	3
2	3	4	5
3	2	2	5
1	1	4	4
4	2	2	4
5	4	4	1
2	2	4	1
2	3	2	3
4	3	3	2
4	4	3	3
3	2	4	5
4	3	4	4
1	1	2	2
5	2	4	2
3	3	2	4
3	1	4	1
5	2	2	3
2	4	4	3
2	1	1	5
4	3	1	5
3	3	4	2
2	4	1	5
3	2	4	1
5	1	3	3
5	3	4	4
5	3	2	5
5	4	2	5
4	4	1	1
1	2	2	3
2	2	1	5
3	3	4	2
5	4	2	5
3	3	2	3
5	4	4	5
4	4	4	3
1	4	2	2
4	4	3	4
2	3	1	1

5	4	3	3
2	3	3	3
5	1	3	3
2	4	4	3
2	4	4	1
1	2	3	2
5	4	2	2
4	1	4	1
2	4	2	5
3	3	3	2
3	1	1	2
1	1	3	1
2	4	3	5
1	4	1	4
1	2	4	5
2	2	4	3
1	4	3	2
5	4	3	2
1	3	4	4
2	3	2	4
4	4	1	2
2	4	2	5
2	2	4	3
1	4	3	3
1	4	2	1
1	1	3	2
3	4	3	4
3	1	4	3
5	4	2	5
4	4	2	2
1	4	2	5
1	4	4	2
1	4	4	1
2	2	4	3
2	4	2	4
2	3	2	1
3	2	3	3
4	3	4	3
2	4	2	2
3	1	4	2
4	3	2	4
5	1	3	2
5	3	3	1
3	3	1	3
3	3	3	2
2	3	4	2

2	1	4	1
1	3	1	2
1	1	1	4
4	3	1	5
4	3	4	5
2	3	4	4
3	3	2	2
2	4	2	1
1	4	2	4
2	4	3	2
3	3	4	4
1	1	4	2
5	4	4	3
1	2	2	3
3	4	3	3
4	3	2	3
2	3	2	1
2	3	3	4
5	3	4	5
4	1	3	3
1	3	3	1
2	3	1	4
4	3	4	4
5	4	2	2
1	3	4	2
2	4	2	4
5	4	1	4
4	3	4	5
3	4	4	5
1	4	2	3
1	4	4	5
5	4	3	5
2	4	2	3
2	3	2	1
5	3	3	5
4	3	1	3
2	4	3	5
1	4	3	4
5	3	2	3
5	4	4	2
2	4	3	3
5	1	2	5
1	2	4	5
1	4	4	5
4	2	3	3
4	1	2	1

5	4	4	4
4	4	4	5
5	2	4	2
3	4	2	5
1	4	4	4
1	1	4	2
4	3	4	4
4	4	3	4
4	4	1	2

Awareness of environmental policy	Propaganda of government	Government subsidies
2	1	0
2	0	0
2	0	0
1	0	0
1	1	0
3	0	0
2	0	1
1	0	0
2	0	1
1	0	0
3	0	0
1	1	1
3	1	0
1	1	1
3	1	1
2	0	1
1	0	1
3	0	1
1	0	1
2	0	0
2	1	0
3	1	0
1	0	1
1	1	0
3	0	0
2	0	0
5	1	0
2	0	0
1	1	1
1	0	0
2	1	0
1	0	0
1	0	0
2	1	1
1	0	1
1	0	1
2	0	1
2	0	1
2	0	1
1	0	1
2	1	0
1	1	0
5	1	1
1	0	0
3	0	1

3	0	0
4	0	1
2	1	0
2	0	0
3	1	1
2	0	0
1	1	1
1	0	0
2	1	0
2	1	1
5	0	1
3	1	0
3	0	0
3	0	1
2	0	1
2	1	0
2	0	0
1	0	0
1	1	1
5	1	0
2	0	1
4	0	0
2	1	0
3	0	0
1	0	0
3	1	0
1	1	0
2	0	1
1	1	1
1	1	1
1	0	1
3	0	0
1	0	0
1	1	0
1	0	1
4	0	0
4	1	1
1	0	1
4	0	1
2	1	0
3	0	0
4	1	1
4	0	0
1	0	1
3	0	0
2	0	1

1	0	1
3	1	1
4	1	1
1	0	1
2	0	0
1	0	0
1	0	0
2	0	0
4	1	0
1	0	1
4	1	1
1	1	0
3	0	0
4	1	0
3	1	0
1	1	0
2	1	1
2	0	0
3	0	0
2	0	1
2	0	0
3	1	1
1	1	0
2	0	1
3	0	0
2	1	0
2	0	0
3	0	0
1	0	0
2	1	0
4	0	0
4	1	1
1	1	0
2	0	0
2	1	1
3	0	0
3	0	0
3	0	1
4	0	1
4	1	1
3	1	1
2	0	1
1	0	0
1	0	0
3	0	1
3	0	0

2	0	0
1	1	1
4	0	0
2	0	1
1	0	0
2	1	1
1	1	1
3	0	0
4	0	0
1	0	0
2	0	1
4	1	1
4	0	1
4	0	1
1	0	0
1	1	0
2	0	1
2	0	1
3	1	0
4	1	0
4	0	0
4	0	1
2	0	1
3	0	0
2	0	0
3	1	1
3	0	0
4	1	0
2	1	0
3	1	1
2	0	0
1	0	0
3	0	0
2	1	0
4	1	0
2	0	0
4	0	1
4	0	0
2	0	1
1	0	1
1	0	0
2	0	1
2	0	0
1	0	0
4	0	1
4	0	0

3	1	0
1	0	1
4	1	1
4	1	1
4	1	1
3	0	0
2	0	0
2	0	1
4	0	0
3	0	1
1	1	0
2	1	0
4	0	0
3	0	1
1	0	1
2	0	0
4	1	1
3	0	0
2	0	1
1	1	1
4	1	0
3	0	1
4	1	0
4	1	0
2	0	1
4	0	1
1	0	1
2	1	0
1	0	0
2	0	1
4	1	0
3	1	0
4	1	0
2	1	0
1	1	1
2	0	1
2	1	1
2	1	1
2	1	1
1	0	1
2	0	0
3	0	1
4	0	1
4	0	1
1	0	1
1	0	1

4	0	1
1	0	1
3	0	0
4	1	0
2	0	1
2	0	1
2	1	0
1	1	1
4	0	0
4	1	1
3	1	1
4	0	1
4	1	1
1	1	1
2	1	1
3	0	1
2	1	0
2	1	1
4	1	0
2	0	1
2	1	1
1	0	1
3	1	1
4	1	1
1	0	0
4	1	1
1	1	1
2	0	0
1	1	0
4	0	1
1	1	0
4	0	1
4	0	1
1	0	1
4	0	0
3	1	1
1	1	1
3	0	0
1	0	0
3	0	0
1	0	0
5	0	0
5	0	1
4	0	0
5	1	1
1	0	1

3	1	1
4	1	0
3	1	1
1	1	1
2	0	1
2	0	0
5	0	1
5	0	1
5	1	0

Decentralization of breeding scale	Cognition of human health	Technical ease of use decentralization
1.264084578	1	0.10915494
1.264084578	1	-0.89084506
-0.735915422	2	0.10915494
-1.735915422	4	-1.89084506
-0.735915422	1	1.10915494
-0.735915422	1	-0.89084506
-0.735915422	5	1.10915494
-0.735915422	1	-0.89084506
0.264084578	1	-1.89084506
-0.735915422	3	-0.89084506
0.264084578	4	-1.89084506
0.264084578	4	1.10915494
-1.735915422	1	-0.89084506
0.264084578	5	-0.89084506
1.264084578	4	-1.89084506
-1.735915422	4	-1.89084506
0.264084578	1	-0.89084506
-1.735915422	4	1.10915494
-1.735915422	1	1.10915494
0.264084578	4	-1.89084506
1.264084578	2	-0.89084506
-0.735915422	2	-1.89084506
-0.735915422	5	-1.89084506
1.264084578	5	-1.89084506
0.264084578	4	1.10915494
-1.735915422	4	-1.89084506
1.264084578	3	-0.89084506
-1.735915422	1	0.10915494
1.264084578	2	-0.89084506
0.264084578	1	-0.89084506
0.264084578	3	0.10915494
-1.735915422	1	1.10915494
0.264084578	3	2.10915494
-1.735915422	1	-1.89084506
1.264084578	4	0.10915494
1.264084578	1	-1.89084506
-0.735915422	2	0.10915494
0.264084578	5	-0.89084506
-0.735915422	1	-0.89084506
-1.735915422	2	2.10915494
0.264084578	3	0.10915494
0.264084578	1	0.10915494
-1.735915422	5	2.10915494
1.264084578	3	-1.89084506
1.264084578	1	-1.89084506

-0.735915422	2	2.10915494
1.264084578	1	2.10915494
-1.735915422	4	-0.89084506
-1.735915422	5	-1.89084506
-0.735915422	5	0.10915494
0.264084578	4	2.10915494
-1.735915422	4	-0.89084506
-0.735915422	2	-1.89084506
1.264084578	4	1.10915494
0.264084578	3	-1.89084506
-0.735915422	3	2.10915494
-1.735915422	3	-1.89084506
-0.735915422	4	1.10915494
-1.735915422	3	-0.89084506
0.264084578	2	-1.89084506
1.264084578	5	-0.89084506
1.264084578	4	-1.89084506
-0.735915422	5	1.10915494
-1.735915422	2	-0.89084506
1.264084578	4	1.10915494
-1.735915422	5	2.10915494
-1.735915422	5	2.10915494
0.264084578	5	2.10915494
-1.735915422	5	1.10915494
-0.735915422	3	-1.89084506
1.264084578	3	0.10915494
0.264084578	4	-0.89084506
-1.735915422	2	1.10915494
0.264084578	3	-0.89084506
1.264084578	5	1.10915494
-1.735915422	5	-0.89084506
0.264084578	4	0.10915494
0.264084578	2	-1.89084506
0.264084578	2	0.10915494
0.264084578	5	0.10915494
0.264084578	3	0.10915494
-1.735915422	4	-1.89084506
0.264084578	5	-1.89084506
-0.735915422	2	0.10915494
1.264084578	5	2.10915494
1.264084578	4	0.10915494
-1.735915422	3	0.10915494
0.264084578	2	0.10915494
0.264084578	3	-1.89084506
-1.735915422	2	0.10915494
0.264084578	5	-1.89084506

1.264084578	3	0.10915494
-1.735915422	2	-0.89084506
-0.735915422	3	0.10915494
0.264084578	5	-0.89084506
-1.735915422	3	0.10915494
1.264084578	2	-0.89084506
1.264084578	3	2.10915494
0.264084578	4	2.10915494
-1.735915422	2	-1.89084506
-1.735915422	5	1.10915494
0.264084578	3	-0.89084506
-0.735915422	3	-1.89084506
0.264084578	2	2.10915494
0.264084578	4	-0.89084506
0.264084578	5	-0.89084506
-1.735915422	2	0.10915494
-1.735915422	3	-1.89084506
-1.735915422	5	-1.89084506
0.264084578	3	0.10915494
0.264084578	3	1.10915494
0.264084578	2	-1.89084506
1.264084578	2	0.10915494
-0.735915422	5	-1.89084506
1.264084578	5	-0.89084506
0.264084578	4	-1.89084506
1.264084578	2	-1.89084506
-0.735915422	4	-1.89084506
1.264084578	3	1.10915494
-1.735915422	5	1.10915494
0.264084578	4	-0.89084506
-0.735915422	2	-0.89084506
1.264084578	5	-0.89084506
0.264084578	5	-0.89084506
1.264084578	3	1.10915494
1.264084578	3	1.10915494
1.264084578	4	-1.89084506
-0.735915422	4	1.10915494
-1.735915422	2	1.10915494
-0.735915422	5	-1.89084506
-1.735915422	5	2.10915494
-1.735915422	4	2.10915494
-0.735915422	5	2.10915494
0.264084578	5	2.10915494
-1.735915422	3	1.10915494
-0.735915422	2	-1.89084506
1.264084578	2	-0.89084506

0.264084578	2	2.10915494
1.264084578	3	-1.89084506
1.264084578	5	-1.89084506
-1.735915422	4	0.10915494
0.264084578	4	0.10915494
-1.735915422	3	1.10915494
-1.735915422	3	1.10915494
-0.735915422	3	0.10915494
0.264084578	5	0.10915494
0.264084578	5	2.10915494
-0.735915422	5	2.10915494
-1.735915422	3	1.10915494
-0.735915422	2	1.10915494
1.264084578	5	-1.89084506
-0.735915422	3	-1.89084506
0.264084578	4	0.10915494
0.264084578	5	-0.89084506
1.264084578	2	0.10915494
-0.735915422	4	2.10915494
0.264084578	5	1.10915494
-1.735915422	5	-0.89084506
-0.735915422	3	-0.89084506
0.264084578	2	1.10915494
-1.735915422	5	-1.89084506
-0.735915422	3	0.10915494
1.264084578	5	0.10915494
-1.735915422	5	2.10915494
0.264084578	4	2.10915494
0.264084578	3	-0.89084506
1.264084578	3	2.10915494
-0.735915422	5	-1.89084506
-1.735915422	5	0.10915494
0.264084578	5	1.10915494
0.264084578	3	2.10915494
1.264084578	3	2.10915494
1.264084578	4	-1.89084506
-0.735915422	2	0.10915494
-0.735915422	5	2.10915494
0.264084578	2	-0.89084506
1.264084578	5	2.10915494
0.264084578	4	0.10915494
1.264084578	5	2.10915494
1.264084578	5	0.10915494
1.264084578	4	-0.89084506
1.264084578	4	1.10915494
0.264084578	5	-1.89084506

1.264084578	3	0.10915494
0.264084578	3	0.10915494
-1.735915422	5	0.10915494
1.264084578	4	0.10915494
1.264084578	2	-1.89084506
-0.735915422	4	-0.89084506
2.264084578	4	-0.89084506
-1.735915422	4	-1.89084506
1.264084578	4	2.10915494
0.264084578	3	-0.89084506
-1.735915422	2	-0.89084506
-1.735915422	2	-1.89084506
1.264084578	5	2.10915494
1.264084578	2	1.10915494
-0.735915422	5	2.10915494
-0.735915422	5	0.10915494
1.264084578	2	-0.89084506
1.264084578	5	-0.89084506
0.264084578	3	1.10915494
0.264084578	4	1.10915494
1.264084578	4	-0.89084506
2.264084578	3	2.10915494
-0.735915422	5	0.10915494
1.264084578	5	0.10915494
1.264084578	4	-1.89084506
-1.735915422	4	-0.89084506
1.264084578	2	1.10915494
-1.735915422	3	0.10915494
1.264084578	3	2.10915494
1.264084578	3	-0.89084506
1.264084578	3	2.10915494
1.264084578	5	-0.89084506
1.264084578	2	-1.89084506
-0.735915422	5	0.10915494
1.264084578	2	1.10915494
0.264084578	4	-1.89084506
-0.735915422	2	0.10915494
0.264084578	5	0.10915494
1.264084578	4	-0.89084506
-1.735915422	4	-0.89084506
0.264084578	5	1.10915494
-1.735915422	4	-0.89084506
0.264084578	4	-1.89084506
0.264084578	2	0.10915494
0.264084578	2	-0.89084506
0.264084578	5	-0.89084506

-1.735915422	1	-1.89084506
0.264084578	3	-0.89084506
-1.735915422	2	1.10915494
0.264084578	3	2.10915494
0.264084578	4	2.10915494
0.264084578	2	1.10915494
0.264084578	4	-0.89084506
1.264084578	2	-1.89084506
1.264084578	2	1.10915494
1.264084578	3	-0.89084506
0.264084578	3	1.10915494
-1.735915422	3	-0.89084506
1.264084578	4	0.10915494
-0.735915422	4	0.10915494
1.264084578	3	0.10915494
0.264084578	3	0.10915494
0.264084578	2	-1.89084506
0.264084578	5	1.10915494
0.264084578	2	2.10915494
-1.735915422	5	0.10915494
0.264084578	5	-1.89084506
0.264084578	3	1.10915494
0.264084578	3	1.10915494
1.264084578	4	-0.89084506
0.264084578	2	-0.89084506
1.264084578	2	1.10915494
1.264084578	2	1.10915494
0.264084578	3	2.10915494
1.264084578	4	2.10915494
1.264084578	2	0.10915494
1.264084578	2	2.10915494
1.264084578	5	2.10915494
1.264084578	4	0.10915494
0.264084578	3	-1.89084506
0.264084578	4	2.10915494
0.264084578	4	0.10915494
1.264084578	5	2.10915494
1.264084578	5	1.10915494
0.264084578	3	0.10915494
1.264084578	2	-0.89084506
1.264084578	5	0.10915494
-1.735915422	4	2.10915494
-0.735915422	4	2.10915494
1.264084578	2	2.10915494
-0.735915422	5	0.10915494
-1.735915422	5	-1.89084506

1.264084578	2	1.10915494
1.264084578	5	2.10915494
-0.735915422	5	-0.89084506
1.264084578	2	2.10915494
1.264084578	4	1.10915494
-1.735915422	4	-0.89084506
0.264084578	5	1.10915494
1.264084578	2	1.10915494
1.264084578	5	-0.89084506

interaction
0.137981072
-1.12610352
-0.080328807
3.282347202
-0.816244245
0.6555866
-0.816244245
0.6555866
-0.499343008
0.6555866
-0.499343008
0.292910725
1.546431661
-0.235258445
-2.390187979
3.282347202
-0.235258445
-1.925399184
-1.925399184
-0.499343008
-1.12610352
1.391502023
1.391502023
-2.390187979
0.292910725
3.282347202
-1.12610352
-0.189483747
-1.12610352
-0.235258445
0.028826136
-1.925399184
0.556995273
3.282347202
0.137981072
-2.390187979
-0.080328807
-0.235258445
0.6555866
-3.661314487
0.028826136
0.028826136
-3.661314487
-2.390187979
-2.390187979

-1.552159667
2.666150331
1.546431661
3.282347202
-0.080328807
0.556995273
1.546431661
1.391502023
1.402065635
-0.499343008
-1.552159667
3.282347202
-0.816244245
1.546431661
-0.499343008
-1.12610352
-2.390187979
-0.816244245
1.546431661
1.402065635
-3.661314487
-3.661314487
0.556995273
-1.925399184
1.391502023
0.137981072
-0.235258445
-1.925399184
-0.235258445
1.402065635
1.546431661
0.028826136
-0.499343008
0.028826136
0.028826136
0.028826136
3.282347202
-0.499343008
-0.080328807
2.666150331
0.137981072
-0.189483747
0.028826136
-0.499343008
-0.189483747
-0.499343008

0.137981072
1.546431661
-0.080328807
-0.235258445
-0.189483747
-1.12610352
2.666150331
0.556995273
3.282347202
-1.925399184
-0.235258445
1.391502023
0.556995273
-0.235258445
-0.235258445
-0.189483747
3.282347202
3.282347202
0.028826136
0.292910725
-0.499343008
0.137981072
1.391502023
-1.12610352
-0.499343008
-2.390187979
1.391502023
1.402065635
-1.925399184
-0.235258445
0.6555866
-1.12610352
-0.235258445
1.402065635
1.402065635
-2.390187979
-0.816244245
-1.925399184
1.391502023
-3.661314487
-3.661314487
-1.552159667
0.556995273
-1.925399184
1.391502023
-1.12610352

0.556995273
-2.390187979
-2.390187979
-0.189483747
0.028826136
-1.925399184
-1.925399184
-0.080328807
0.028826136
0.556995273
-1.552159667
-1.925399184
-0.816244245
-2.390187979
1.391502023
0.028826136
-0.235258445
0.137981072
-1.552159667
0.292910725
1.546431661
0.6555866
0.292910725
3.282347202
-0.080328807
0.137981072
-3.661314487
0.556995273
-0.235258445
2.666150331
1.391502023
-0.189483747
0.292910725
0.556995273
2.666150331
-2.390187979
-0.080328807
-1.552159667
-0.235258445
2.666150331
0.028826136
2.666150331
0.137981072
-1.12610352
1.402065635
-0.499343008

0.137981072
0.028826136
-0.189483747
0.137981072
-2.390187979
0.6555866
-2.016948462
3.282347202
2.666150331
-0.235258445
1.546431661
3.282347202
2.666150331
1.402065635
-1.552159667
-0.080328807
-1.12610352
-1.12610352
0.292910725
0.292910725
-1.12610352
4.775305271
-0.080328807
0.137981072
-2.390187979
1.546431661
1.402065635
-0.189483747
2.666150331
-1.12610352
2.666150331
-1.12610352
-2.390187979
-0.080328807
1.402065635
-0.499343008
-0.080328807
0.028826136
-1.12610352
1.546431661
0.292910725
1.546431661
-0.499343008
0.028826136
-0.235258445
-0.235258445

3.282347202
-0.235258445
-1.925399184
0.556995273
0.556995273
0.292910725
-0.235258445
-2.390187979
1.402065635
-1.12610352
0.292910725
1.546431661
0.137981072
-0.080328807
0.137981072
0.028826136
-0.499343008
0.292910725
0.556995273
-0.189483747
-0.499343008
0.292910725
0.292910725
-1.12610352
-0.235258445
1.402065635
1.402065635
0.556995273
2.666150331
0.137981072
2.666150331
2.666150331
0.137981072
-0.499343008
0.556995273
0.028826136
2.666150331
1.402065635
0.028826136
-1.12610352
0.137981072
-3.661314487
-1.552159667
2.666150331
-0.080328807
3.282347202

1.402065635
2.666150331
0.6555866
2.666150331
1.402065635
1.546431661
0.292910725
1.402065635
-1.12610352
